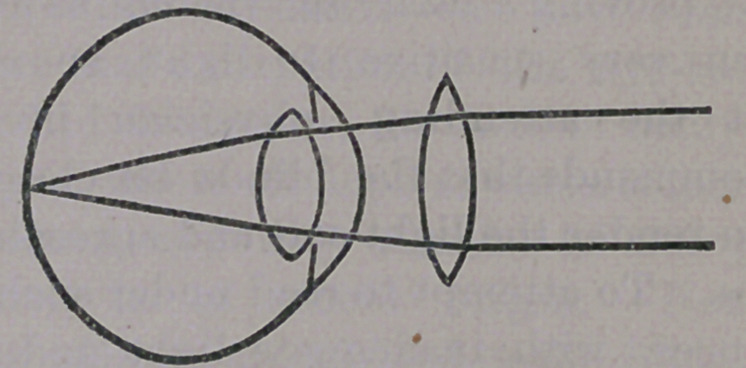# Near and Long Sightedness

**Published:** 1874-10

**Authors:** 


### NEAR AND LONG SIGHTEDNESS.

Near-sightedness, or *myopia,* is a condition of the eye in which it becomes necessary to hold the book very close to the eye, in order to be able to distinguish the letters. Persons with myopic eyes are utterly unable to see faces across the room, and will pass their most intimate acquaintances upon the walk without being able to recognize them. When the difficulty first presents itself to the the child, it is discovered that his eye becomes tired while reading at school, that he is unable to see the figures clearly upon the blackboard, and that the letters run together and blur, in such a manner as to be absolutely painful to his eyes. Often the child will come from school complaining of pain in his eyes, and of frontal headache. This is the beginning of *myopia,* and depends upon a change in the shape of the eyeball, which, in health, should be nearly round. When afflicted in the manner described, the ball becomes elongated or egg-shaped, as here illustrated :—

causing the parallel rays of light, coming from distant objects, to fall to a focus before reaching the retina, as shown in the above cut, where the rays are seen to cross each other before they reach that membrane. The longer this diameter of the eye is, the greater will be the confusion of the image, and the closer to the eye will the object be held.

A popular delusion is that near-sighted people will be able to see when they grow old, without glasses, and they are often congratulated that their old age will afford them

"young eyes." Another fallacy is that the near-sighted eye is a *strong* one, and that for this reason, they will bear more use and abuse than a normal eye. The contrary is the truth, for, when the difficulty comes on in youth and gradually increases in severity, so that the book is brought closer and closer to the eye, it becomes a very serious trouble, leading often to what the ophthalmic surgeon terms *posterior staphyloma,* a condition that produces complete blindness.

The only remedy for near-sightedness is to have some competent oculist test the eyes, and prescribe such concave lenses as are exactly suited to them. These glasses should be neither too strong nor too weak, but just of sufficient strength to overcome the amount of deformity present. The manner in which the lens rectifies this defect in vision will be understood by almost every school-boy, who will tell you that concave lenses disperse the rays of light, rendering parallel rays divergent, a condition of affairs needed in this case before us. But it requires skill, and suitable apparatus to enable one to prescribe the power and precise form of the lens necessary to compensate for the exact amount of elongation of the ball, in every case, in order to disperse the rays of light sufficiently to have them fall in their proper position upon the retina, at once rendering the image perfect and the vision clear and distinct. And here let us caution the myopic against purchasing spectacles from the traveling peddler or the dealer in "Brazilian Pebbles," who stops at the principal hotel in the city, and by ingenious advertising —securing testimonials from the prominent physicians, for instance, in payment for a set of his "pebbles," fitted to the eyes of the willing practitioner—induces you to purchase a pair of glasses at a price ranging from fifteen to seventy-five dollars, depending largely upon your gullability. Glasses should only be prescribed after a suitable ophthalmoscopic and test-glass examination, and never by the spectacle peddler or the jeweler. We are daily called upon to examine weak and dim eyes that are rendered so by the use of badly-fitting glasses.

The reverse of the condition of the eyes above referred to, is that known as long or over-sightedness, and described in ophthalmic works as *hypermetropia.* It will be seen from the figure below (fig. 1.) that the eyeball instead of being too long, is too flat, so that the rays of light would come to a focus behind the retina, as illustrated. To overcome this requires great effort of *accommodation,* which at last so tires the muscles engaged in this office, as to give rise to "weak eyes;" the patient complaining of his eyes feeling tired and fatigued after protracted reading. This trouble continues until reading becomes absolutely impossible, when relief is found in a lens shaped as below given,—

which converges the parallel rays sufficiently to enable the eye to focus them at the proper point upon the retina, at once rendering the confused and blurred vision perfectly distinct.

So great is this deformity of the eye that it is not unfrequently the case that young children, from five to ten years of age, are compelled to wear convex glasses of a strength suitable for a person of eighty years of age. We have met with many children whose eyes were nearly ruined for want of suitable aids to vision, by reason of the ignorance and prejudice of parents who would not permit them to wear such glasses as were suited to their eyes, simply because they were "old enough" for grandpa or grandma to see from. Deprived of these aids, over-sighted children soon begin to squint, caused by the great effort set forth in trying to *accommodate* the eye, at short range of vision. It has been discovered that very many of the cases of *strabismus* or "cross-eyes" are caused by need of convex glasses in aiding the *hypermetropic* eye to properly focus the image upon its retina.

In view of the many defects of vision depending upon some fault in the refractive power of the eye, only two of which we have here described, are we not right in insisting that an ophthalmic surgeon should be consulted in all such instances, rather than trust to your own judgment or that of the dealer in spectacles?

## Figures and Tables

**Figure f1:**
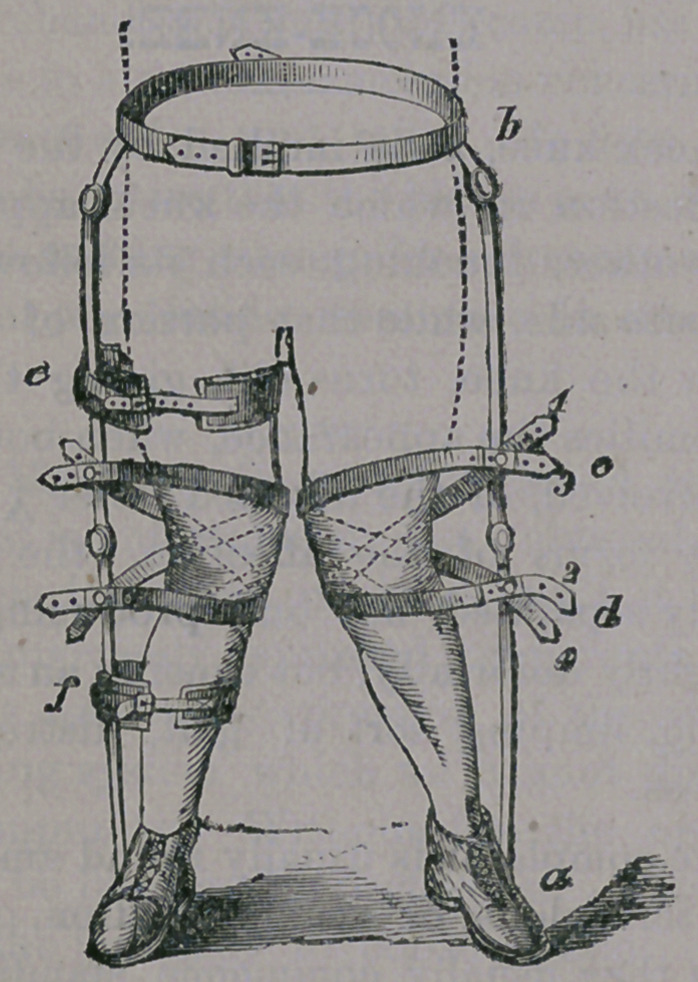


**Figure f2:**
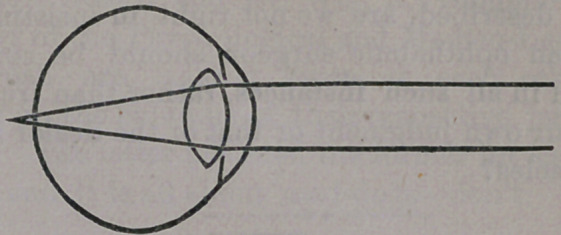


**Figure f3:**